# Design of an integrable double-sided optoplasmonic gyroscope via a bent hybrid structure

**DOI:** 10.1038/s41598-024-61279-w

**Published:** 2024-05-06

**Authors:** Jalal Gholinejad, Kambiz Abedi

**Affiliations:** Electronic Department, Shahid Beheshti University, Tehran, Iran

**Keywords:** Gyroscope, Optical, Surface plasmon polaritons, Hybrid structure, Applied optics, Optical materials and structures, Optical techniques

## Abstract

This paper presents an optoplasmonic gyroscope that employs a novel bent-hybrid structure, and works double-sided. The proposed device is integrable without moving parts, and simply has a robust configuration. The detection mechanism is based on surface plasmon polaritons (SPPs), and the sensor consists of a laser, a bent-metal layer, and a photo-detector (PD). Based on the simulations, the proposed gyroscope provides significant characteristics of a measurement range of ± 45000°/s, an optical sensitivity of 6.025 µ/(°/s), a total sensitivity of 2.41 µA/W(°/s), an ultra-high resolution ability of 16.598 µ°/s, and an accuracy of 99.999%. The dimensions are 5 × 5 × 5 µm^3^, and the measurement time is 1 ms. The operational wavelength is at visible range of λ = 630 nm. Additionally, the effects of various parameters, including metal material, metal thickness, and laser wavelength, on the gyroscope performance are comprehensively studied.

## Introduction

After the discovery of laser in 1966, a window was opened for the field of optical gyroscopes^1,2^. Optical gyroscopes are frequently used in various applications such as navigation, aerospace industry, robotics, and smart phones^1,3^. Optical gyroscopes are categorized in three types of fiber optic gyroscopes (FOGs), ring laser gyroscopes (RLGs), and optoelectronic gyroscopes^1,4,5^. The duty of a gyroscope is to determine the angular velocity of a rotating object^1,6–9^. In fact, optical gyroscopes provide noteworthy features like high speed, high accuracy, and high reliability in optical wavelengths^1,4,10–12^. However, these sensors have some shortages including big size and high resolution^1,12–15^. Consequently, it is needed to investigate some solutions to remove the mentioned issues.

Wang Xiaowei et al.^4^ introduced a photonic crystal (PC) FOG which had an optimized birefringence-stress stability. The main approach in this work was to implement several rectangles into the fiber cladding. It was declared that this mechanical buffer may help to improve the performance of FOG. Shuangchao Ge et al.^16^ demonstrated a method to control the winding speed of fiber optic coil (FOC). This can improve tension shortages, and may help FOG to achieve higher robustness. Parham P. Khial et al.^17^ proposed a nanophotonic-based gyroscope. In fact, this work introduced an interferometer with two optical loops, and tried to improve the performance by decreasing phase mismatch. However, the obtained dimensions in this work were big.

Plasmons demonstrate useful characteristics like high sensitivity and small dimensions in optical frequencies, and these may be employed in various optoelectronic-based devices^18–20^. Since 2011, researches have been conducted on the application of surface plasmon polaritons (SPPs) in gyroscope structures. Yu-Chu M. Li et al.^21^ provided a new interferometer with SPP oscillation. In this research, using a plasmonic section that has high sensitivity, the performance of the gyroscope was optimized. The SPP section was used to control the phase and adjust the equality of two optical paths which increased the sensitivity. Wei Li et al.^22^ used a plasmonic waveguide to take advantages of SPPs. Consequently, the sensitivity was controlled by an electrical signal.

Using an active SPP loop, Tong Zhang et al.^23^ proposed a hybrid gyroscope. In this sensor, a plasmonic path was employed as the loop of gyroscope. This technique increased the sensitivity and reduced the loss; however, the signal-to-noise ratio (SNR) was decreased. Yang-Yang Wang et al.^24^ examined noise due to spontaneous emission in the previous gyroscope. This paper studied the spectrum of this device, and increased the SNR. Tong Zhang et al.^25^ designed a SPP-based component to inject light into the gyroscope optical loop. In this research, the SPP coupling coefficient contributed to optimize the sensitivity of the gyroscope; nevertheless, the reported size was big.

Accordingly, in continue to our ongoing project on optical gyroscopes^6–9^, we provide a novel hybrid structure to be used as SPP-based integrable optical gyroscope which solves the discussed drawbacks of conventional devices. The idea of using SPPs for measuring angular velocity is established based on the plasmonics waves’ characteristics, including high sensitivity and sub-wavelength confinement (small dimensions). While previous works focused on the possibility of using SPP in the gyroscope structure rather than designing an integrable structure. However, this research provides a novel structure which satisfies the characteristics that are needed for industrial sensors. Moreover, the sensing methodology is based on a creative technique which employs SPP on a bent metallic layer.

The introduced sensor is double-sided, meaning it can detect the direction of rotation. A hybrid bent configuration is used, and the design has no moving parts. The proposed structure operates all-optical, and provides significant features including small dimensions, high sensitivity, low resolution, low response time, and low required optical power at visible wavelength of λ = 630 nm. Besides, the designing procedure is explained so that can be used as a pattern in this art of study. Furthermore, the effect of various parameters on the performance of the introduced gyroscope is discussed, and it can guide engineers for the designing of SPP-based sensors.

In forward, the article is organized as: in Sect. "Model of proposed gyroscope", the structure and the mechanism of the gyroscope are modeled. Subsequently, in Sect. "Results and discussions", the results are discussed. Afterwards, Sect. "Comparison" compares the gyroscope with previous works. Finally, in Sect. "Conclusion", the conclusion is done.

## Model of proposed gyroscope

Here, the mechanism of the designed gyroscope is described, and the associated mathematical model is carried out. Afterwards, the parameters of the structure are introduced. The simulations are done with LUMERICAL FDTD software.

### Gyroscope structure

**Figure 1:**
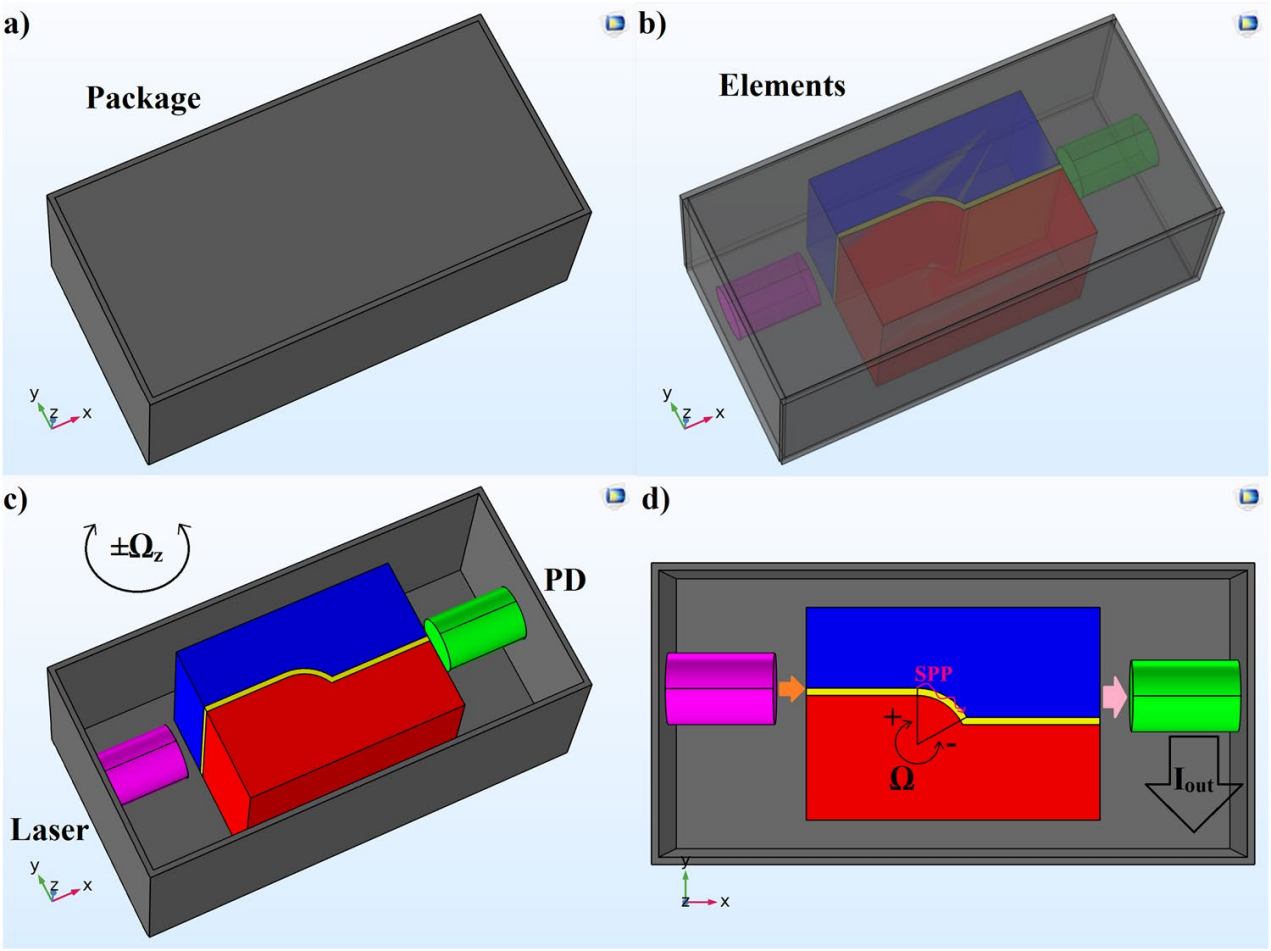
The illustration of (**a**) packed sensor, (**b**) internal elements of the device, (**c**) assembly of the components, and (**d**) gyroscope mechanism.

In Fig. 1, the designed gyroscope is illustrated, where an input optical continues wave (CW) ray using a commercial laser is introduced to the bent-hybrid structure. Next, SPP waves pass the bent path, and introducing an angular velocity changes the angle of bent virtually. Next, the length against SPP is modulated proportional to the input angular velocity, ± Ω. Consequently, the amount of absorbed power is altered. Then, the transmitted optical power is measured via a balanced photo-detector (PD), PDB230A. An anticlockwise angular velocity reduces the SPP path and absorbed power, while increases the transmitted optical power. A clockwise one extends the metal bent, and adds up the absorption, resulting lower transmission. It should be notified that in this design there is no need for mechanical parts, and the readout circuit is simply to detect the output current of PD. Actually, in this design the optical loop is based on an integrated hybrid-metallic-bent structure instead of fiber optics, where this makes plasmonic features available for the gyroscope performance. Moreover, the sensing technique does not include any mechanical mechanism, and is established based on SPP to achieve its high sensitivity and small dimension characteristics.

### Measurement mechanism

**Figure 2:**
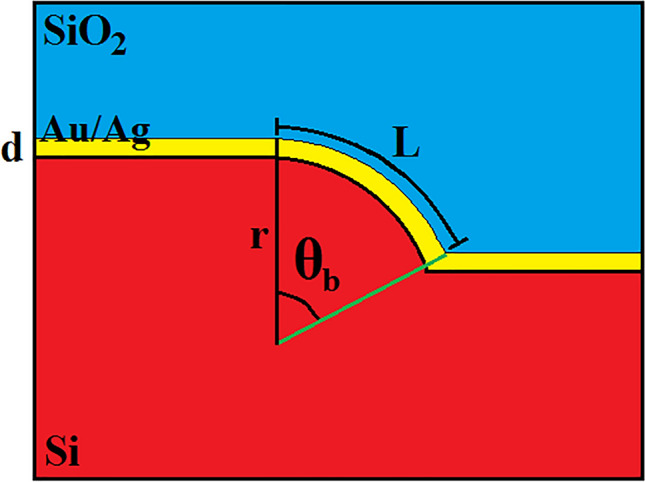
The presentation of bent-hybrid layer and SPP path.

The sensor orbits around the center of bent, and the optical path can be modeled as follow^1,26,27^:

1 $$L={L}_{0}\pm \Delta L={L}_{0}\pm \frac{2\pi r}{360}\Delta t\Omega ,$$

where $L$ is the SPP path, ${L}_{0}$ is the initial bent path, $\Omega$ is the angular velocity, $\Delta t$ is time period, and $r$ is the radius of bent. In fact, clockwise angular velocity provides more available metallic structure and more SPP absorption, while anticlockwise angular velocity lessens the bent. As seen in Fig. 2, the bent angle, ${\theta }_{b}$, can be achieved as:

2 $${\theta }_{b}=\frac{360}{2\pi r}L.$$

Using Eqs. (1) and (2) the relation of bent angle modulation by angular velocity can be achieved:

3 $${\theta }_{b}=\frac{360}{2\pi r}{L}_{0}\pm \Delta t\Omega .$$

Assuming a limit for ${\theta }_{b}$ governs to the input angular range as:

4 $$0\le {\theta }_{b}\le 90\to {\Omega }_{min}\le \Omega \le {\Omega }_{max}.$$

**Figure 3:**
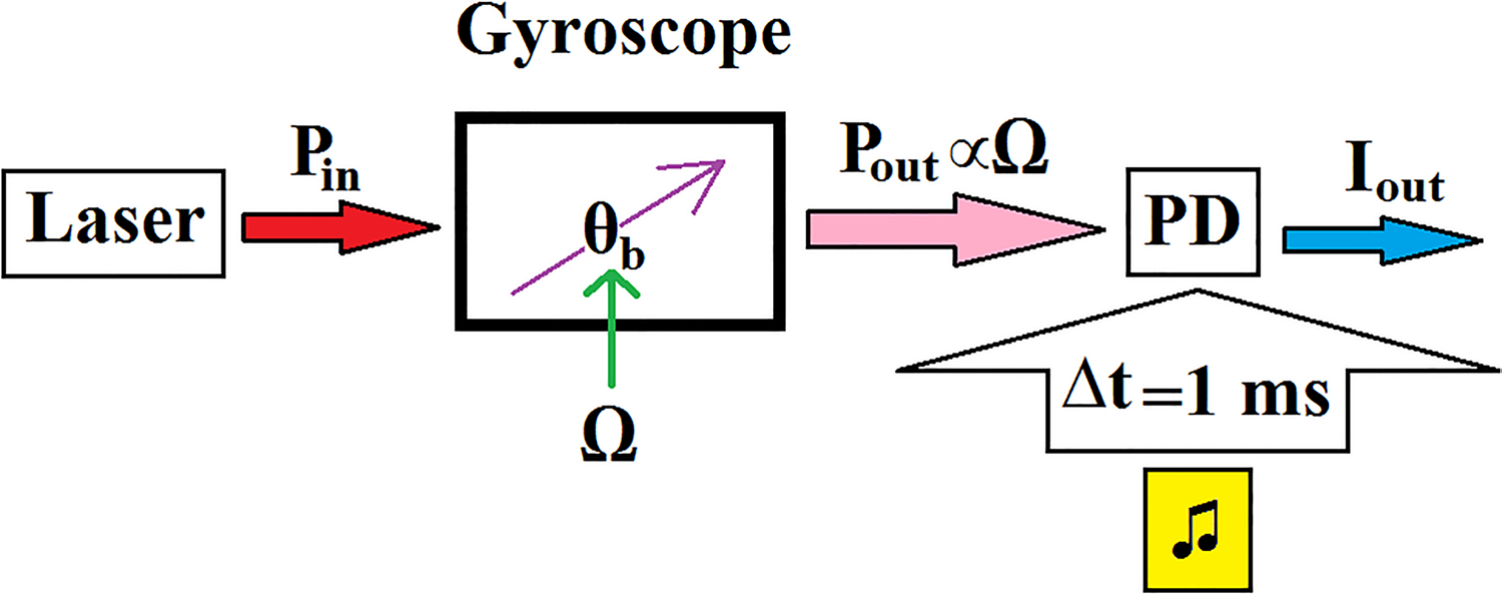
The introduced approach of angular velocity measurement.

The discussed procedure is shown in Fig. 3, where the input angular velocity is measured each $\Delta t=1 \,ms$ of time period. The employed approach uses an input optical power via a Laser, where its beam incidences to the designed structure. The input angular velocity modulates the bent angle, ${\theta }_{b}$, of the hybrid-metallic structure, subsequently the transmitted optical power is changed in proportion to the input angular velocity. Finally, the optical power is read with a commercial PD, where its output current can be measured. Moreover, the design parameters are listed in Table 1.

**Table 1:** The parameters of the gyroscope.

	Explanation	Range
r	The bent radius	1 μm
${{\varvec{\theta}}}_{0}$	The initial bent angle	45 °
${{\varvec{L}}}_{0}=\frac{2{\varvec{\pi}}{\varvec{r}}{{\varvec{\theta}}}_{0}}{360}$	The initial bent SPP path	785.4 nm
Metal material	Gold and silver	Au, Ag
d	The metal layer thickness	20, 50, 150 nm
λ	The optical wavelength	410, 550, 630 nm

## Results and discussions

**Figure 4:**
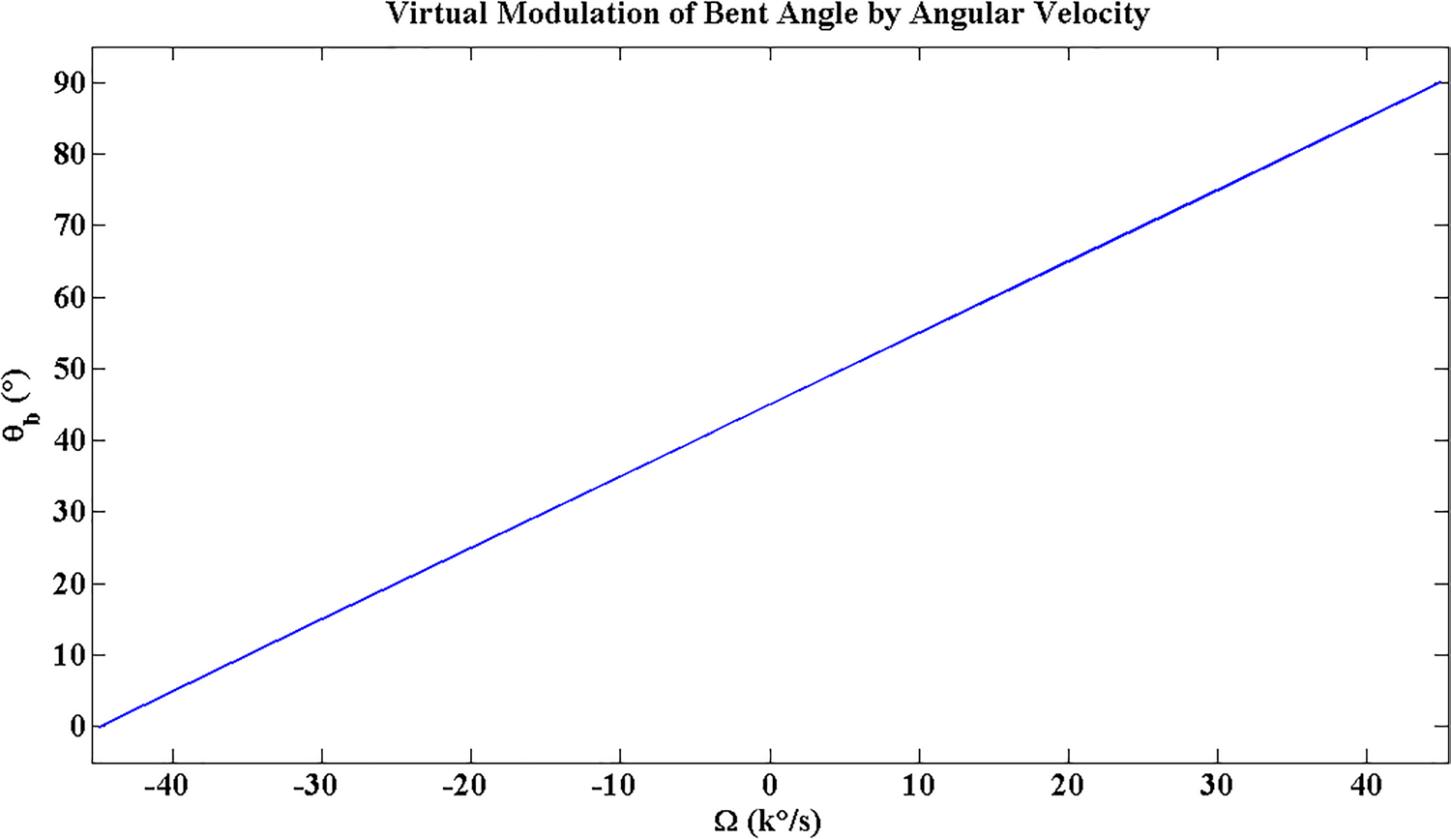
The modulation of bent angle by input angular velocity.

Using Eqs. (3), (4), and the data in Table 1, the virtual change of bent angle vs input angular velocity can be obtained. As shown in Fig. 4, the angular velocity range is $-45000^\circ /s\le \Omega \le +45000^\circ /s$.

**Figure 5:**
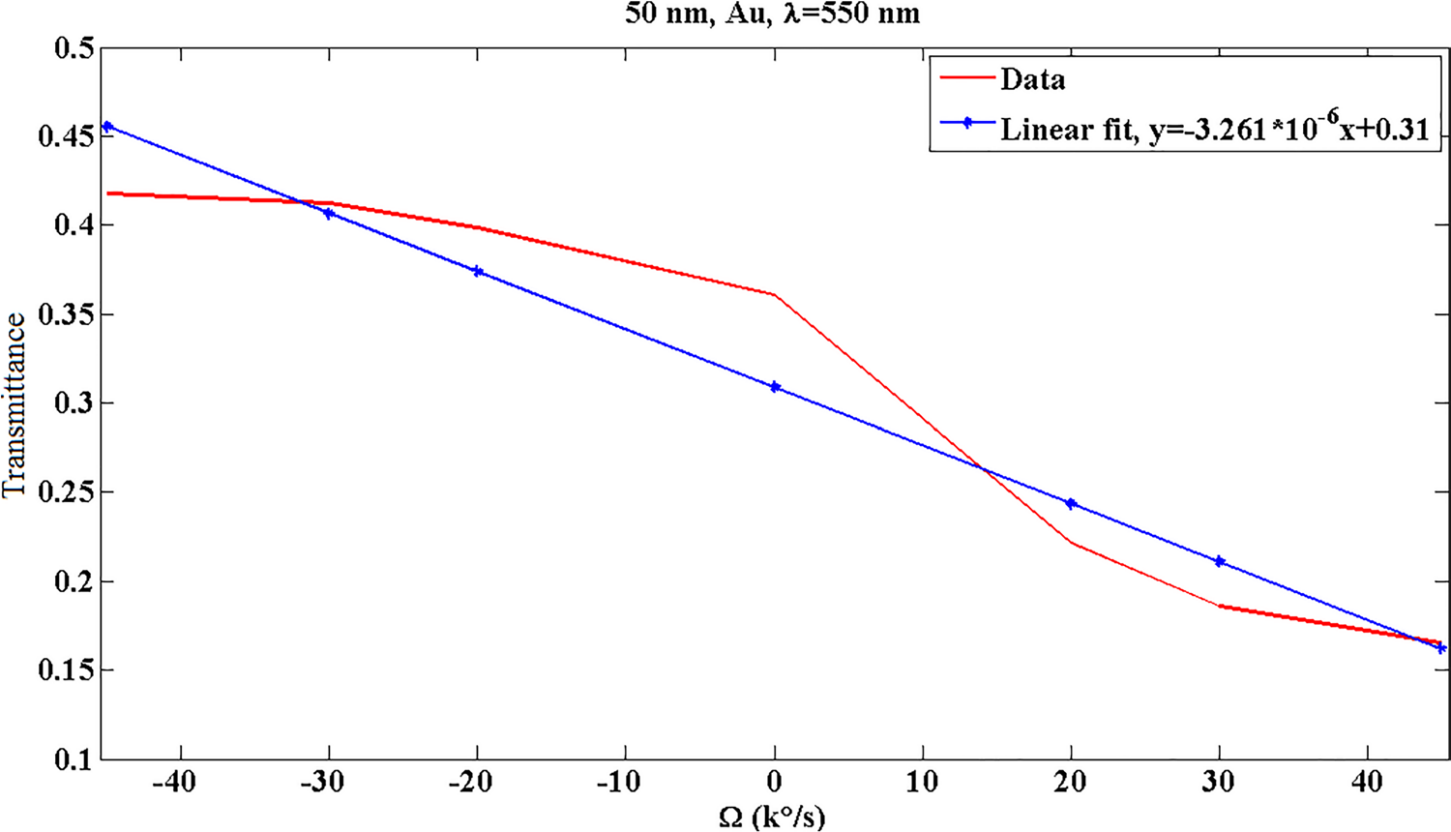
The transmitted optical power and fitted data for 50-nm Au at λ = 550 nm. R^2^, the square of the correlation, is 0.9233.

**Figure 6:**
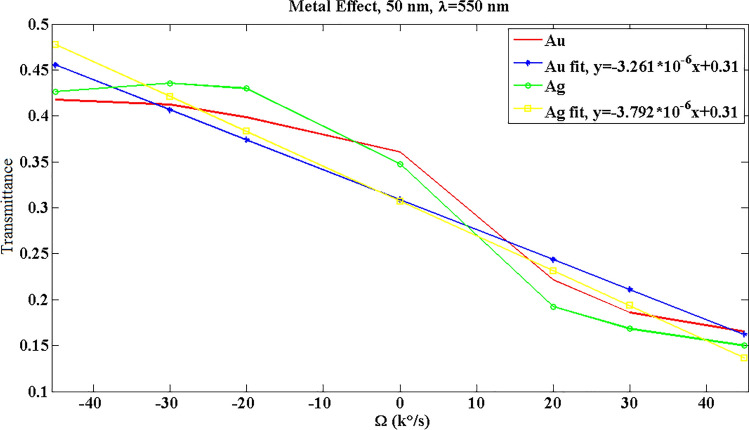
The effect of metallic material on the optical transmittance response of system for d = 50 nm and λ = 550 nm. R^2^ for Au and its fit is 0.9233, and for Ag R^2^ equals to 0.9135.

Figure 5 displays the transmitted optical power for 50-nm Au at λ = 550 nm, where the data are approximated using first-order fits. The slope of this curve indicates the optical sensitivity of gyroscope. This proposes the change of transmitted optical power due to the input angular velocity. In Fig. 6, the effect of SPP material on the gyroscope results is studied. It is obvious that silver shows a higher slope (sensitivity); however, there is an unfavorable behavior in the range of − 45000 °/s to − 20000 °/s. Ag bent does not provide a function in this region, where for instance a transmittance amount of 0.43 may show two different angular velocities. Therefore, Au can be a reliable selection for this design.

**Figure 7:**
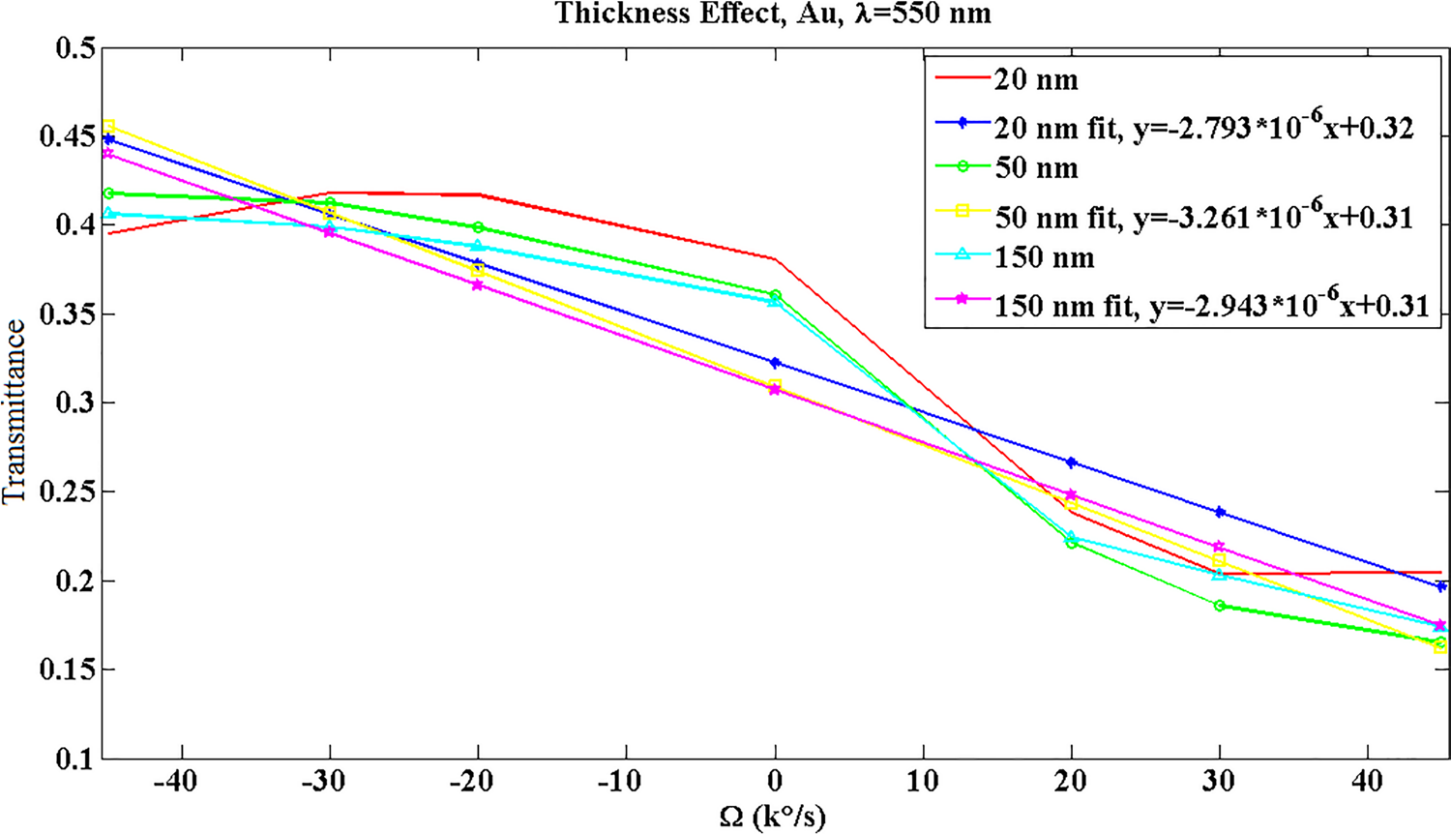
The effect of metal thickness on the optical transmittance response for Au and λ = 550 nm. R^2^ is 0.8399, 0.9233, and 0.9224 for 20 nm, 50 nm, and 150 nm curves, respectively.

Figure 7 demonstrates the thickness effect on the transmittance results. As seen, a thickness of 20 nm has the same nonlinearity problem as mentioned about Ag, and this may be due to the symmetric/asymmetric plasmonic mode phenomenon^28^. Moreover, in this thickness a lower sensitivity is available as shown in fit data. Besides, a thickness of 150 nm has a lower sensitivity than 50 nm. Therefore, a 50-nm Au is chosen in continue.

**Figure 8:**
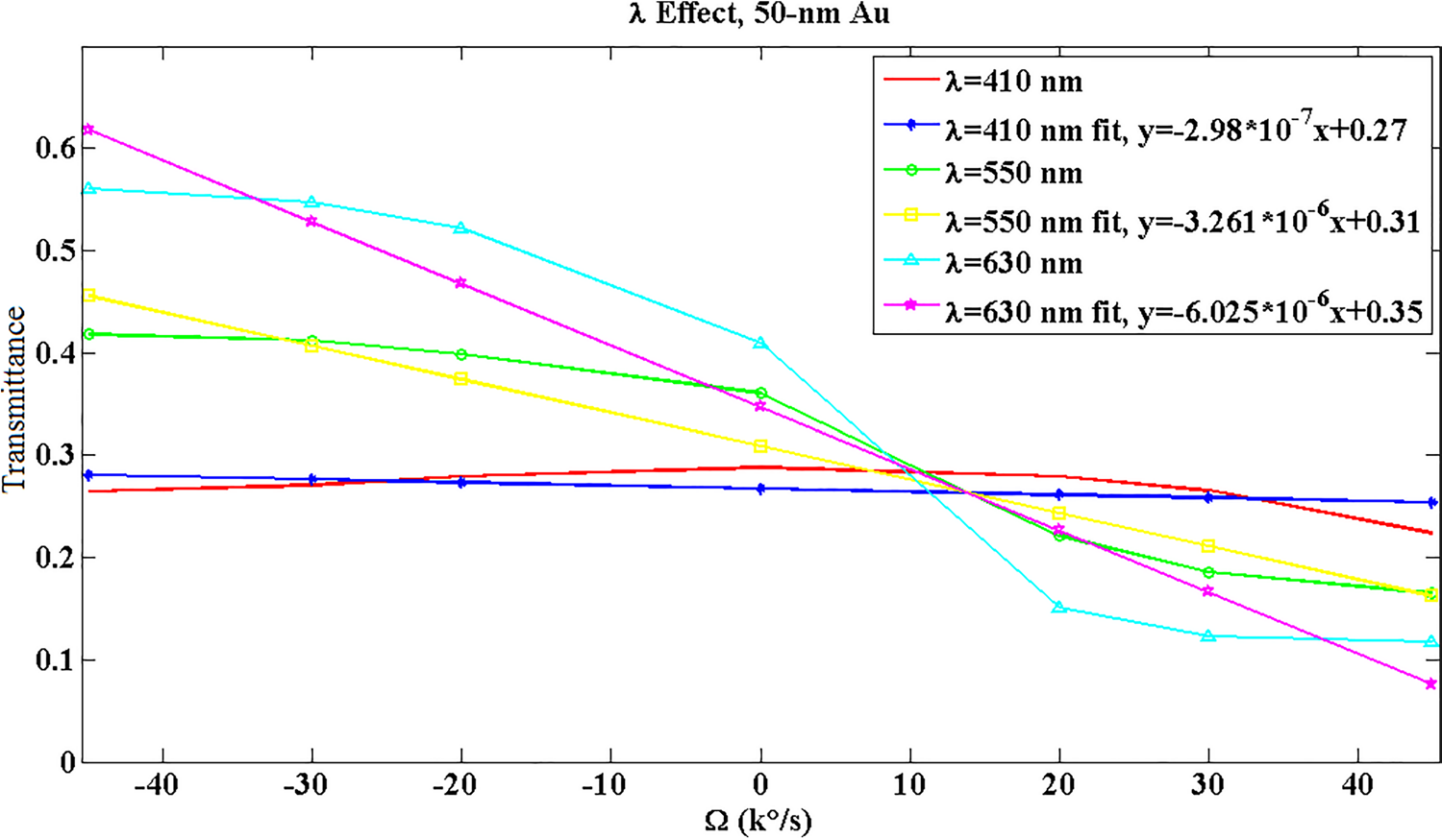
The optical wavelength effect on the optical transmittance response for 50-nm Au. The associated square of correlation coefficients are ${R}_{410 nm}^{2}=0.2235$, ${R}_{550 nm}^{2}=0.9233$, and ${R}_{630 nm}^{2}=0.9242$.

**Figure 9:**
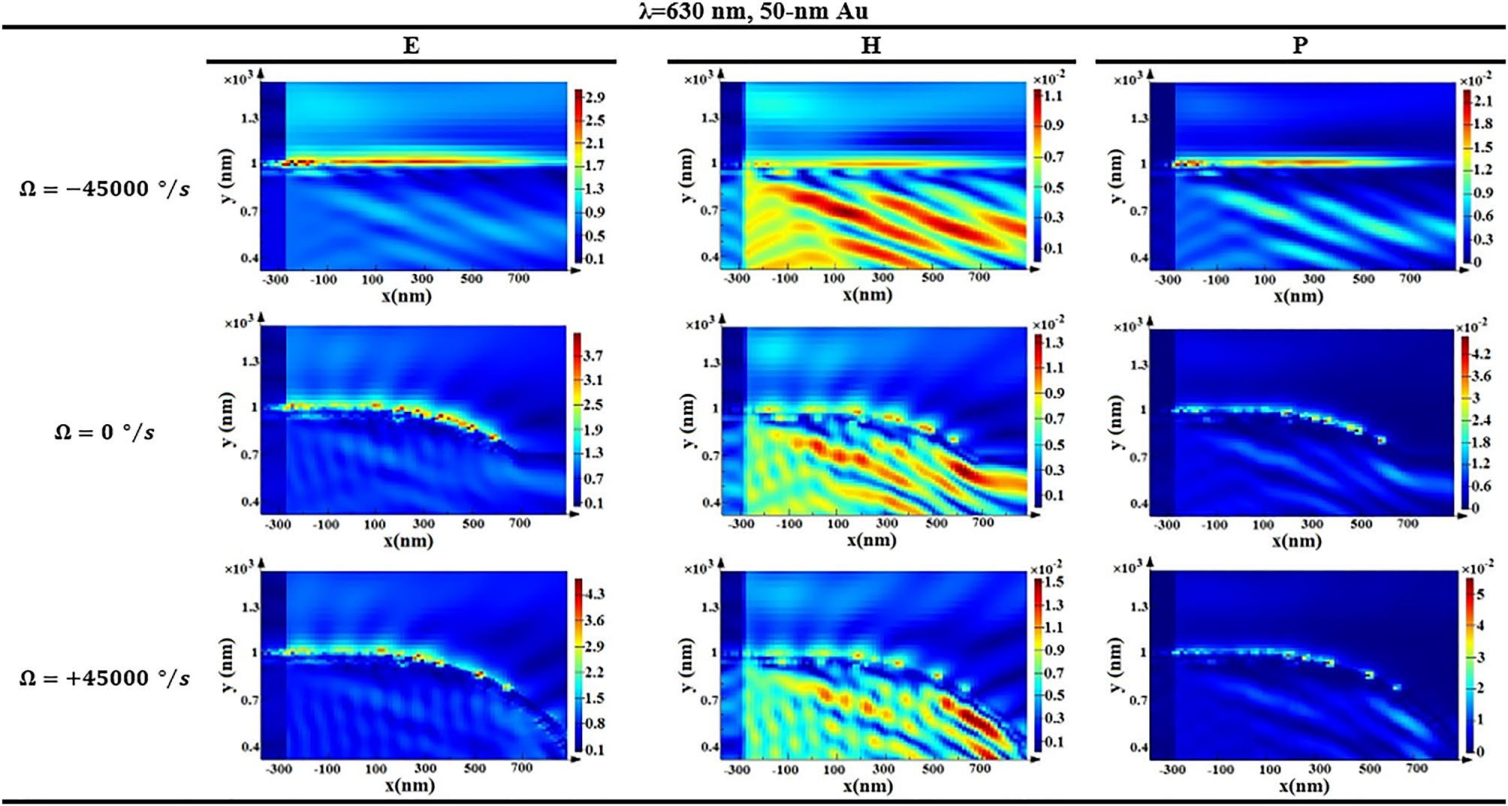
Distribution of electric field, magnetic field, and optical power in a cross section for various angular velocities.

As illustrated in Fig. 8, lower wavelengths have nonlinearity problems, and conclude to lower sensitivities. While a wavelength of λ = 630 nm proposes a higher sensitivity. Moreover, the lasers in this range are commercial, and PD provides a higher responsivity in this wavelength^27,29^. Hence, λ = 630 nm can be the optimized amount for the best performance of the gyroscope. Besides, in Fig. 9, the cross-section profiles of the electrical, magnetic, and optical power are visualized for various angular velocities. As seen, for anticlockwise angular velocities there is a lower bent. While for clockwise angular velocities the bent is extended, and there is more SPP and power absorption. The absorption of optical power is done by metallic layer, and it is observable that the associated bent is changed with input angular velocity. Consequently, the amount of transmitted power is varied. Moreover, the number of the power point on the metallic bent is more with higher intensities for clockwise angular velocities, and it means that SPP waves are excited more in this case. Subsequently, the optical power absorption is more for clockwise angular velocity, and the transmission is less. The mentioned action can be observed in GIF S1, too.

**Figure 10:**
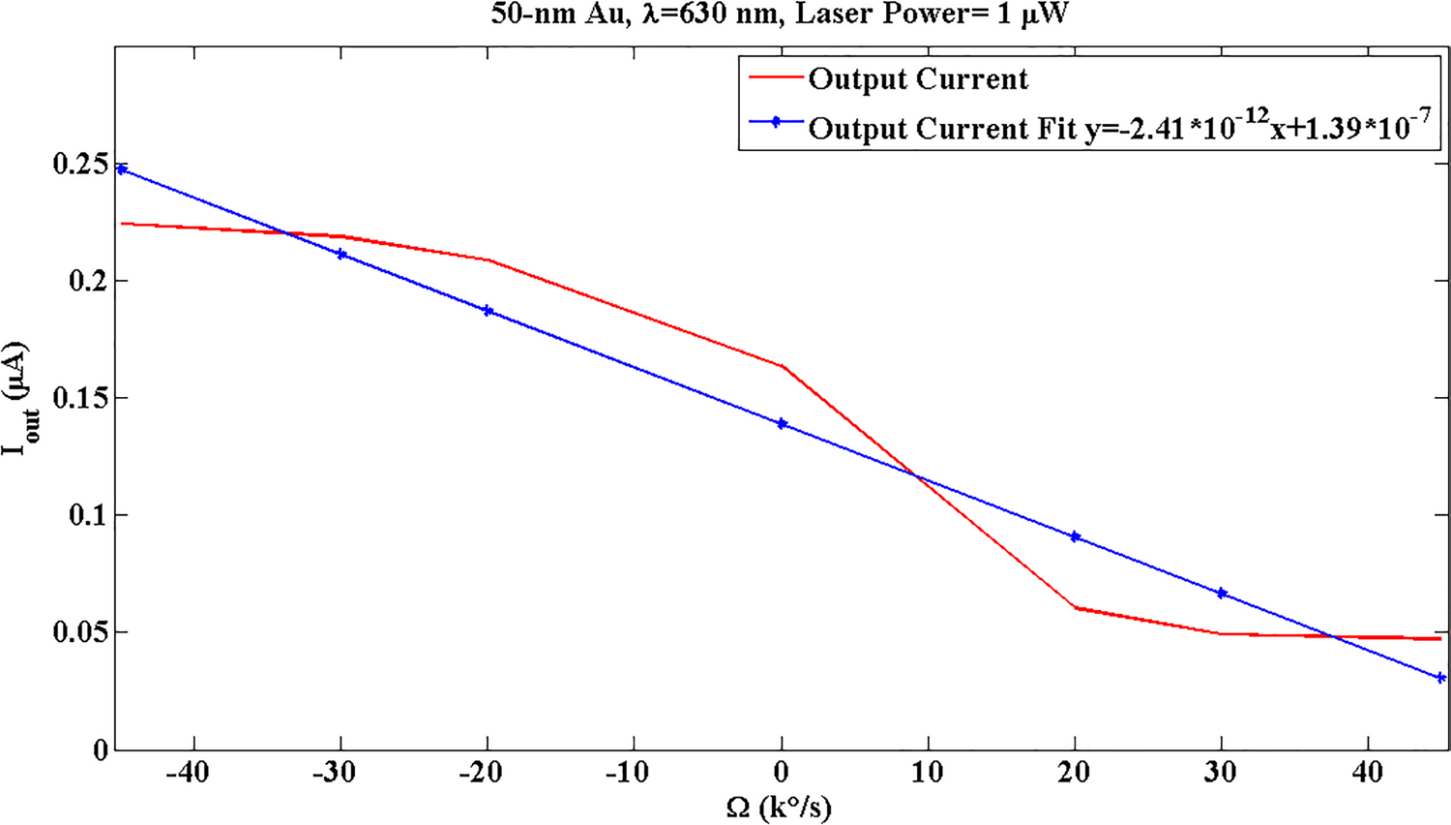
The output current vs input angular velocity for 50-nm Au at λ = 630 nm with a laser power of 1 µW. The related square of correlation coefficient of output current and its linear fit is R^2^ = 0.9241.

The optical results in Fig. 8 can be modeled by the slope of fit data as:

5 $${P}_{transmitted}={S}_{O}\Omega ,$$

where ${S}_{O}$ is the optical sensitivity (the slope), and the results are available for 50-nm Au @ λ = 630 nm in Table 2. Noise equivalent power (NEP) for PDB230A is 12 pW, and the minimum reflected power for the designed system is 11.77% at Ω = 45,000 °/s (Fig. 8), so the minimum required laser power is 102 PW. Moreover, the PD resolution, ${P}_{Res}={10}^{-10} W$, can be used in Eq. (5) to Achieve the resolution of sensor, ${\Omega }_{Res}$. Furthermore, by the responsivity of PD, R = 0.4 A/W, the output current can be calculated as Ref.^27^:

6 $${{I}_{out}=RP}_{transmitted}=R{S}_{O}\Omega ={S}_{t}\Omega ,$$

where ${S}_{t}$ is the total sensitivity and the slope of fit curve of Fig. 10.

**Table 2:** The parameters of the designed gyroscope.

Parameter	Explain	Value
R	Responsivity of PD	0.4 A/W
∆Ω	Angular velocity range	$\pm 45000^\circ /s$
Dimensions	–	5 × 5 × 5 µm^3^
λ	Laser wavelength	630 nm
${{\varvec{S}}}_{{\varvec{O}}{\varvec{p}}{\varvec{t}}}$	Optical sensitivity	6.025 µ/(°/s)
${{\varvec{S}}}_{{\varvec{t}}}$	Total sensitivity	2.41 µA/W(°/s)
${{\varvec{P}}}_{{\varvec{m}}{\varvec{i}}{\varvec{n}}}$	Minimum laser power	102 pW
∆t	Response time	1 ms
${{\varvec{\Omega}}}_{{\varvec{R}}{\varvec{e}}{\varvec{s}}}$	Gyroscope resolution	16.598 µ°/s

## Comparison

In Table 3, the parameters of the proposed gyroscope are compared with previous works. The introduced gyroscope has a new bent-hybrid structure based on SPP technique. The angular velocity range is 79 times wider than other designs, and the operational wavelength is λ = 630 nm. The size is 11 times less, and its dimensions are suitable for integration with CMOS devices. Resolution amount is ultra-low (providing high resolution ability), and the sensitivities are favorably high. Moreover, as discussed the required optical power is ultra-low, thus the power consumption can be expected to be low. In fact, some of previous works use mechanical sensing methods, and some use fiber optics loop. While the introduced gyroscope contains no mechanical parts, and employs an integrable SPP bent as the optical loop. This leads to achieve features like high sensitivity and small size with a simple sensing technique. Moreover, the properties of the designed sensor is discussed comprehensively, and the design procedure is provided so that can be assumed as a pattern for future works.

**Table 3:** The comparison of the results of the designed gyroscope vs previous works.

	Optical sensitivity	Total sensitivity	Dimensions	Operational Wavelength (nm)	Resolution	Performance range (°/s)	Double sided
Bent hybrid structure (proposed work)	6.025 µ/(°/s)	2.41 µA/W(°/s)	5 × 5 × 5 µm^3^	630	16.598 µ°/s	± 45,000	Yes
MOEMS electrostatic comb-drive actuator^6^	0.1051 nm/(º/s)	–	405 × 405 × 40 µm^3^	1599	–	± 570	Yes
Passive PC ring^30^	–	–	279 μm^2^	1514	–	–	No
Crystalline waveguide^31^	–	0.0278º/s	3.6 mm	1550	–	–	Yes
MEMS U-beam ring^32^	–	0.6 mV/(°/s)	6 × 6 × 0.15 mm^3^	600–1200	–	± 200	Yes
Multi-gap SPP waveguide^33^	–	–	6 cm^2^ × 8 µm	1550	833.33 µ°/s	–	Yes

## Conclusion

In this paper, an optical gyroscope based on a novel bent-hybrid structure and surface plasmon polaritons is introduced. The designed sensor is integrable and has small dimensions of 5 × 5 × 5 µm^3^. Moreover, significant characteristics of measurement range of ± 45000 °s, optical sensitivity of 6.025 µ/(°/s), total sensitivity of 2.41 µA/W(°/s), ultra-high resolution ability of 16.598 µ°/s, and measurement time of 1 ms are achieved. The operational wavelength is at visible range of λ = 630 nm, and the structure has no moving parts. Furthermore, a comprehensive survey on the effects of various parameters, including metal material, metal thickness, and laser wavelength, on the device results is done (Supplementary GIF S1).

### Supplementary Information

Supplementary GIF S1.

## Data Availability

All required data are provided in the paper.
