# Supplementary figures and images for: Design of an integrable double-sided optoplasmonic gyroscope via a bent hybrid structure
Source: Sci Rep. 2024 May 6;14:10408. doi: 10.1038/s41598-024-61279-w (PMC11074308; doi:10.1038/s41598-024-61279-w)

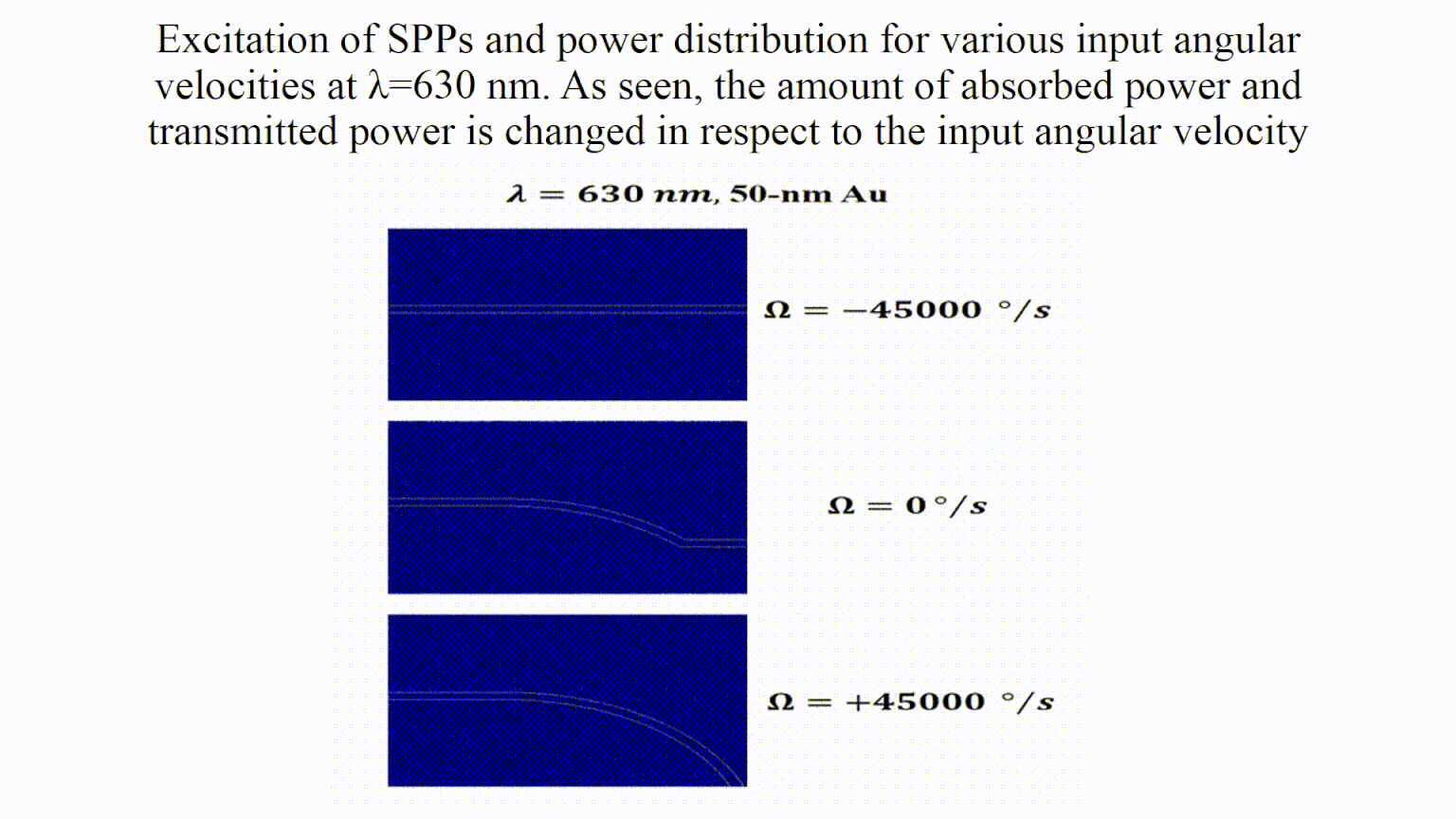

Supplement: Supplementary file 1 — Supplementary GIF S1. [file 41598_2024_61279_MOESM1_ESM.gif]
